# Response of the *Propylea japonica* Microbiota to Treatment with Cry1B Protein

**DOI:** 10.3390/genes14112008

**Published:** 2023-10-27

**Authors:** Fengchao Diao, Yarong Li, Xueke Gao, Junyu Luo, Xiangzhen Zhu, Li Wang, Kaixin Zhang, Dongyang Li, Jichao Ji, Jinjie Cui

**Affiliations:** 1 Zhengzhou Research Base, National Key Laboratory of Cotton Bio-Breeding and Integrated Utilization, School of Agricultural Sciences, Zhengzhou University, Zhengzhou 450001, China; diaofengchao@126.com (F.D.); 15036138389@163.com (X.G.); luojunyu1818@126.com (J.L.); hnnydxjc@163.com (J.J.); 2 National Key Laboratory of Cotton Bio-Breeding and Integrated Utilization, Institute of Cotton, Chinese Academy of Agricultural Sciences, Anyang 455000, China; 15638749495@163.com (Y.L.); zhuxiangzhen318@163.com (X.Z.); wangli08zb@126.com (L.W.); zhangkaixin@caas.cn (K.Z.); hzaulidongyang@163.com (D.L.)

**Keywords:** Bt protein, *Propylea japonica*, 16S rRNA, endophyte, biosafety

## Abstract

*Propylea japonica* (Thunberg) (Coleoptera: Coccinellidae) is a dominant natural enemy of insect pests in farmland ecosystems. It also serves as an important non-target insect for environmental safety evaluations of transgenic crops. Widespread planting of transgenic crops may result in direct or indirect exposure of *P. japonica* to recombinant *Bacillus thuringiensis* (Bt) protein, which may in turn affect the biological performance of this natural enemy by affecting the *P. japonica* microflora. However, the effects of Bt proteins (such as Cry1B) on the *P. japonica* microbiota are currently unclear. Here, we used a high-throughput sequencing method to investigate differences in the *P. japonica* microbiota resulting from treatment with Cry1B compared to a sucrose control. The results demonstrated that the *P. japonica* microbiome was dominated by Firmicutes at the phylum level and by Staphylococcus at the genus level. Within-sample (α) diversity indices demonstrated a high degree of consistency between the microbial communities of *P. japonica* treated with the sucrose control and those treated with 0.25 or 0.5 mg/mL Cry1B. Furthermore, there were no significant differences in the abundance of any taxa after treatment with 0.25 mg/mL Cry1B for 24 or 48 h, and treatment with 0.5 mg/mL Cry1B for 24 or 48 h led to changes only in *Staphylococcus*, a member of the phylum Firmicutes. Treatment with a high Cry1B concentration (1.0 mg/mL) for 24 or 48 h caused significant changes in the abundance of specific taxa (e.g., *Gemmatimonades*, *Patescibacteria*, *Thauera*, and *Microbacterium*). However, compared with the control, most taxa remained unchanged. The statistically significant differences may have been due to the stimulatory effects of treatment with a high concentration of Cry1B. Overall, the results showed that Cry1B protein could alter endophytic bacterial community abundance, but not composition, in *P. japonica*. The effects of Bt proteins on endophytes and other parameters in non-target insects require further study. This study provides data support for the safety evaluation of transgenic plants.

## 1. Introduction

Transgenic crops have been commercially planted for over 20 years, beginning in 1996, and transgenic crop cultivation not only protects biodiversity but also confers economic benefits, and can therefore reduce poverty in some regions [1]. Numerous studies have predicted the necessity of cultivating genetically modified crops across the globe in the future; as a result, studies associated with genetically modified crops are highly significant [2]. Several transgenic crops are commonly cultivated at present, including corn, soybean, rapeseed, papaya, beet, eggplant, and potato. Transgenic cotton serves as an exemplary case of a widely adopted transgenic crop [3,4]. Nonetheless, the large-scale cultivation of genetically modified crops faces a crucial potential issue: the possibility of adverse effects on non-target organisms with key ecological functions [5]. Consequently, many countries require environmental safety risk assessments and regulatory approval before the commercial cultivation of genetically modified crops is possible [6,7]. It is therefore imperative to assess the risks of genetically modified crops to non-target species [8].

Transgenic cotton is one of the most important export cash crops in the world. Lepidopteran insects are among the 15 most economically damaging pests that affect cotton yield [9,10]. Bt protein is a kind of protein secreted by *Bacillus thuringiensis*. Genes encoding the *B. thuringiensis* (Bt) series of crystalline biotoxins are often inserted into cotton to enable resistance to these pests [11]. Bt toxins are highly effective against a wide range of crop pests, including lepidopteran, coleopteran, and dipteran insects, in addition to some invertebrates such as nematodes [12,13]; they are therefore widely used as biopesticides throughout the world. More than 100 different types of Bt proteins, including subspecies of the types Cry1-Cry78, Cyt1-Cyt3, and Vip1-Vip3, have been identified globally [14]. Recent research on Bt proteins has focused on their ecological roles and expression in agricultural and natural environments, as well as the evolution of resistance mechanisms among target pests [15]. Over the past few decades, several studies have demonstrated that Bt transgenic cotton is not harmful to the environment or to humans [16]. Furthermore, it helps to control major pests and reduces the need for pesticides [17]. To date, the safety of Bt proteins in non-target predatory insects has primarily been assessed in terms of the impacts on growth and development. For instance, one study examined the effects of transgenic rice producing the Bt protein Cry1Ab on the biological characteristics and functional responses of the stinkbug *Cyrtorhinus lividipennis (Reuter) (Hemiptera: Miridae)*, an important predator insect [18]. Another study explored whether Bt protein has a negative impact on the population density, predation capacity, or relative abundance of a non-target spider species [19]. A separate study assessed the effects of Cry1B on *Propylea japonica* growth, development, enzyme activity, and expression of genes related to detoxification and metabolism. The results suggest that Cry1B protein has little or no effect on the biological characteristics of *P. japonica*. Genes related to enzyme activity and detoxification are differentially expressed at high-concentration stimulation [20]. 

Microbial community diversity in insects, especially in the gut, is a prominent area of research at present [21]. The methods and measurement techniques employed in this field are becoming increasingly sophisticated and mature [14]. Symbiotic bacteria and micro-organisms play crucial roles in various aspects of insect growth and development, reproduction, and population fitness [22]. Over time, insects have formed close, mutually beneficial relationships with a variety of symbiotic bacteria through natural selection and long-term co-evolution [23]. The microbiota assists the insect host by enhancing disease resistance, adaptability, toxin metabolism, and defense functions. Compared with the host, they are more sensitive to environmental changes [24]. Symbiotic bacteria and other micro-organisms actively participate in various physiological and biochemical regulatory processes and metabolic pathways within insects [23]. For instance, they influence insect reproductive and mating behaviors by altering the flow of material information [22]. Additionally, they contribute to pathogen resistance and can synthesize and transform essential nutrients [24,25], such as critical vitamins and amino acids that cannot be synthesized by the host itself [26,27]. Studies conducted to date have yielded conflicting results regarding whether Bt proteins affect the microbial communities of non-target organisms. For example, although one study showed that the earthworm intestinal microbiome was not significantly altered by consumption of Bt protein compared to the control group [28], another study found slight differences in the relative dominant bacteria in worms fed Cry2Ab compared to control worms [29]. In general, the potential effects of the release and accumulation of Bt proteins on the microbial communities of non-target insects are of increasing concern [30]. Therefore, the potential risks of GM crops must be further assessed using high-throughput techniques.

Predators play a crucial role in all types of ecosystems. *P. japonica* (Thunberg) (Coleoptera: Coccinellidae) is a common predator that feeds on cotton bollworm larvae, aphids, and planthoppers in farmlands [20]. *P. japonica* often comes into contact with Cry proteins, either indirectly, through consumption of prey exposed to transgenic Bt cotton, or directly, through transgenic cotton pollen [31]. As a result, *P. japonica* can serve as an indicator of potential adverse ecosystem effects in transgenic crop fields [32,33,34,35]. However, only limited research has been conducted to assess the impacts of Bt proteins on the microbial communities of non-target insects. Examining the non-target insect microbiota could enhance our understanding of unique biochemical mechanisms and pathways in insects, which have important implications for pest control and natural enemy protection. Here, we treated *P. japonica* larvae at two developmental stages (second and third instar) with varying concentrations of Cry1B (0.25, 0.5, and 1.0 mg/mL) for two durations (24 and 48 h). We then studied changes in the commensal microflora of the *P. japonica* larvae. This study provides new data support for the environmental safety evaluation of genetically modified crops.

## 2. Materials and Methods

### 2.1. Insects

*P. japonica* were collected from the East Field Test Base of Cotton Research at the Chinese Academy of Agricultural Sciences (CAAS), Anyang City, Henan Province, China (36°5′34.8″ N, 114°31′47.19″ E). Individuals were transferred to cages (35 × 35 × 35 cm) that were kept in a climate-controlled chamber at 25 ± 1 °C with 70 ± 10% relative humidity under a 16/8 h light/dark cycle. The *P. japonica* population was raised in the laboratory for five generations prior to the start of experiments. *P. japonica* were fed pea aphids (*Acyrthosiphon pisum*), which were raised in a climate chamber at 24 ± 1 °C with 60 ± 10% relative humidity under a 16/8 h light/dark cycle and fed *Vicia faba* seedling. 

### 2.2. Reagents

Sucrose (CAS: 57-50-1) was purchased from Beijing Solebo Technology Co., Ltd. (Beijing, China). Cry1B was purchased from the Plant Protection Biotechnology Laboratory of the Institute of Plant Protection, CAAS (Anyang, China). Sucrose was diluted to a working concentration of 2 mol/L. Cry1B was dissolved in the 2 mol/L sucrose solution to final concentrations of 0.25, 0.5, and 1.0 mg/mL [36,37,38]. All solutions were stored at 4 °C prior to use.

### 2.3. Insect Sample Collection and Processing

*P. japonica* egg masses were collected and placed in a Petri dish. Eggs were then incubated at 25 ± 1 °C with 70 ± 10% relative humidity under a 14/10 h light/dark cycle until they hatched. Hatched larvae were removed with a brush and placed into individual 1.5 mL centrifuge tubes with holes, then enough pea aphids were provided for them to eat so that they could survive to the second and third instars. Second- and third-instar *P. japonica* larvae were treated with one of several concentrations of Cry1B solution (C1: 0.25, C2: 0.5, or C3: 1.0 mg/mL) for two durations (24 or 48 h). The control group was treated with a 2 mol/L sucrose solution for the same durations. Collected larvae were snap-frozen in liquid nitrogen, then stored at −80 °C prior to further processing. Each biological replicate comprised 10 larvae per treatment group and there were three biological replicates. 

### 2.4. DNA Extraction and PCR Amplification

Microbial DNA was extracted from larvae using the E.Z.N.A.^®^ soil DNA Kit (Omega Bio-tek, Norcross, GA, USA) following the manufacturer’s protocol. DNA concentration and purity were measured with a NanoDrop 2000 UV-vis spectrophotometer (Thermo Fisher Scientific, Wilmington, NC, USA), and DNA quality was assessed with 1% agarose gel electrophoresis. The V3–V4 hypervariable regions of the bacterial 16S rRNA gene were amplified with the 338F (5′-ACTCCTACGGGAGGCAGCAG-3′) and 806R (5′-GGACTACHVGGGTWTCTAAT-3′) primers. PCR was conducted on a GeneAmp 9700 instrument (ABI, Waltham, MA, USA) with the following thermocycling program: denaturation for 3 min at 95 °C; 27 cycles of 30 s at 95 °C, 30 s at 55 °C, and 45 s at 72 °C; and then maintained at 72 °C for 10 min. PCR reactions were performed in technical triplicate in a 20 μL reaction mixture containing 4 μL of 5× FastPfu Buffer, 2 μL of 2.5 mM dNTPs, 0.8 μL each of forward and reverse primers (at 5 μM), 0.4 μL of FastPfu Polymerase, and 10 ng of template DNA. The resulting PCR products were extracted from a 2% agarose gel and purified using the AxyPrep DNA Gel Extraction Kit (Axygen Biosciences, Union City, CA, USA). The purified DNA was then quantified using a QuantiFluor™-ST fluorometer (Promega, Madison, WI, USA) following the manufacturer’s protocol.

### 2.5. Illumina Sequencing

A PE 2 × 300 bp Illumina MiSeq library was constructed from the purified PCR products following the manufacturer’s instructions (Illumina, San Diego, CA, USA). Sequencing was performed on the MiSeq PE300 platform. 

### 2.6. Sequencing Data Processing

The raw FASTQ files were demultiplexed and quality-filtered with Trimmomatic, then merged using FLASH Cs6, accessed on 10 September 2022 (http://www.usadellab.org/cms/?page=trimmomatic). The following criteria were used for quality control and merging: (1) reads were truncated at any site with an average quality score < 20 over a 50 bp sliding window; (2) primers were allowed 2 nucleotide mismatches; (3) reads containing ambiguous bases were removed; and (4) sequences were merged on overlap regions > 10 bp.

Sequences were clustered into operational taxonomic units (OTUs) at a threshold of 97% similarity using UPARSE v7.1 (http://www.drive5.com/uparse/). Chimeric sequences were identified and removed using UCHIME v4.1. Taxonomy was assigned for each OTU with the RDP Classifier algorithm (https://ngdc.cncb.ac.cn/databasecommons/database/id/237) using the Silva (SSU123) 16S rRNA database and a confidence threshold of 70%. The abundance-based coverage estimator (ACE) and Shannon α-diversity indices were calculated in the vegan package of R v3.6.3, which was also used to generate rank abundance and species accumulation curves. Rarefaction curves were also generated from OTU counts in mothur.

### 2.7. Statistical Analysis

Statistical analyses were conducted in SPSS v18.0 (SPSS Inc., Chicago, IL, USA). Significant differences were determined with one-way analysis of variance (ANOVA) and post hoc pairwise comparisons.

## 3. Results

### 3.1. Sequencing Data Assessment 

**Figure 1:**
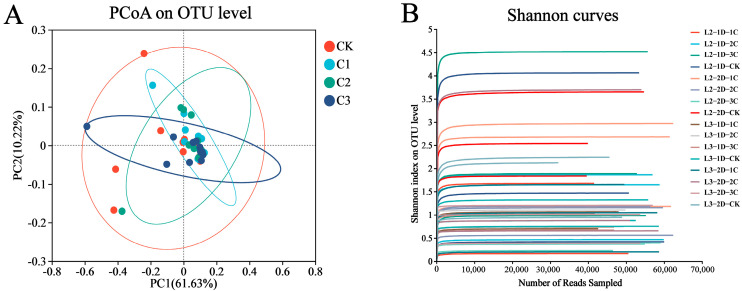
Bacterial community dynamics among *Propylea japonica* treated with several concentrations of the Bt protein Cry1B for two lengths of time: (**A**) Principal coordinate analysis of high-throughput sequencing data using weighted UniFrac values. Ellipses indicate 95% confidence regions for a single treatment group. (**B**) Rarefaction curves based on species abundance data. C1: 0.25 mg/mL Cry1B; C2: 0.5 mg/mL Cry1B; C3: 1.0 mg/mL Cry1B; and CK: sucrose control group.

In total, Illumina MiSeq obtained more than 20,000 high-quality readings. After removing chimeras and sequences with a count of 1, representative unique sequences were selected and clustered into OTUs at 97% similarity. Most samples had between 200 and 300 OTUs, although the maximum was 605 OTUs. The average merged sequence length was 428 nucleotides. The community diversity, richness, and coverage were high for each sample (Table 1), demonstrating the suitability of these data for further analyses. The rarefaction curves, which reflected the microbial diversity of each sample at a range of sequencing depths, reached the asymptote prior to the true number of reads per sample (Figure 1B), demonstrating that the sequencing depth was sufficient to accurately represent community diversity in the sequenced samples. 

### 3.2. Effects of Cry1B on P. japonica Microbial Community Diversity

**Figure 2:**
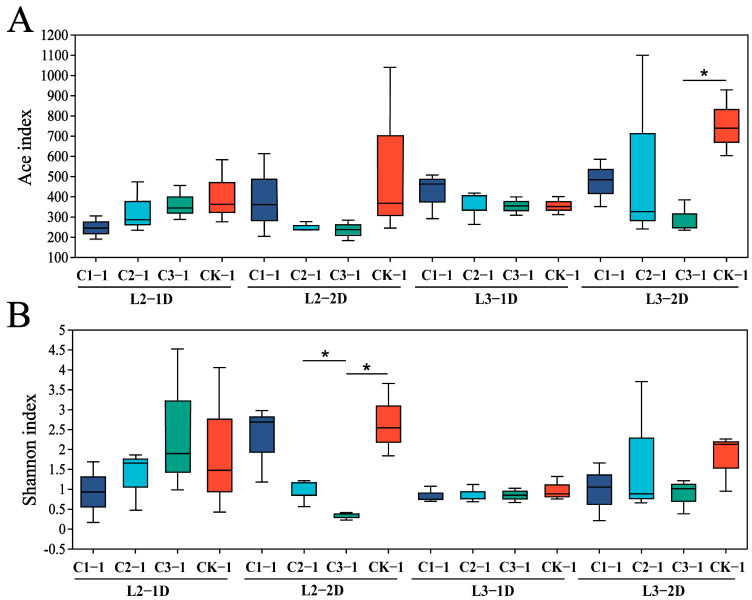
Analysis of *Propylea japonica* microbial communities using 16S rDNA gene sequencing and α-diversity indices. Assessment of differences between samples using the (**A**) abundance-based coverage estimator (ACE) and (**B**) Shannon diversity indices. * *p* ≤ 0.05 (Student’s *t*-test). C1 represents 0.25 mg/mL Cry1B, C2 represents 0.5 mg/mL Cry1B, C3 represents 1.0 mg/mL Cry1B, and CK represents sucrose control group.

There were no significant differences between the Shannon or ACE indices of larvae treated with any concentration of Cry1B compared to those treated with the sucrose control for 24 h. When the treatment time was 48 h, the Shannon index of the second-instar larvae treated with 0.5 mg/mL (*p* = 0.039) or 1.0 mg/mL (*p* = 0.011) Cry1B was significantly different from that treated with sucrose. Among the third-instar larvae, the ACE index was significantly different among those treated with 1.0 mg/mL Cry1B compared to the control (*p* = 0.011) (Figure 2).

A principal coordinate analysis (PCoA) was conducted on weighted UniFrac distance values to assess between-sample (i.e., β) diversity. Integrating the same treatment time at different ages with the same treatment concentration, the *P. japonica* samples treated with Cry1B were more aggregated than those treated with sucrose, indicating that the OTU of *P. japonica* samples at different ages fed with the protein had more similar species composition (Figure 1A). 

### 3.3. Effects of Cry1B on P. japonica Microbiota at the Phylum Level 

**Figure 3:**
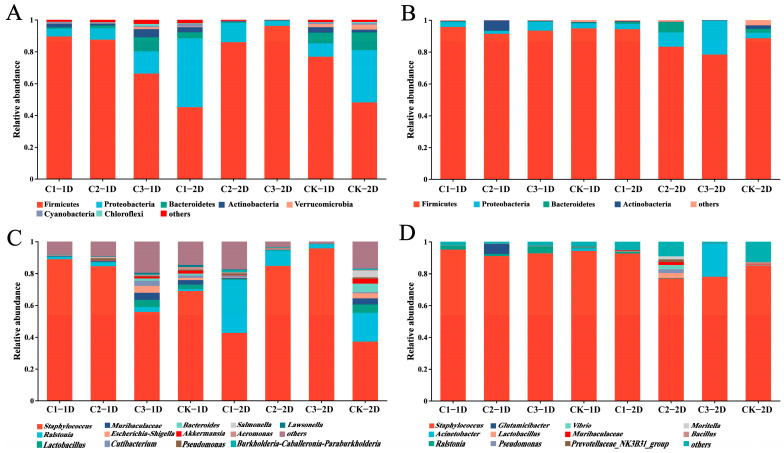
Bacterial abundance changes among *Propylea japonica* treated with several concentrations of the Cry1B for two lengths of time: (**A**,**B**) Relative abundance of bacterial taxa at the phylum level in (**A**) second- and (**B**) third-instar *P. japonica* larvae. (**C**,**D**) Relative abundance of bacterial taxa at the genus level in (**C**) second- and (**D**) third-instar *P. japonica* larvae. All taxa with a relative abundance of <0.01 in all samples were merged into a single taxon called “other”. C1: 0.25 mg/mL Cry1B; C2: 0.5 mg/mL Cry1B; C3: 1.0 mg/mL Cry1B; and CK: sucrose control group.

**Figure 4:**
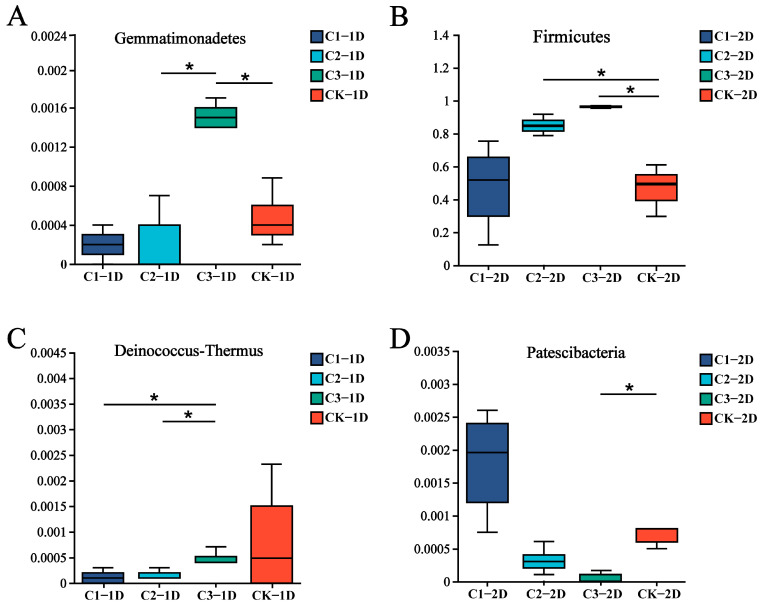
Relative abundance of endophytic bacterial phyla among *Propylea japonica* treated with Cry1B or a sucrose control: (**A**,**B**,**D**) Differences in the bacterial communities of second-instar larvae treated for (**A**) 24 h (2L1D) or (**B**,**D**) 48 h (2L2D). (**C**) Differences in the bacterial communities of third-instar larvae treated for 24 h (3L1D). Data are shown only for phyla with statistically significant differences between groups. * *p* ≤ 0.05 (one-way analysis of variance). C1: 0.25 mg/mL Cry1B; C2: 0.5 mg/mL Cry1B; C3: 1.0 mg/mL Cry1B; and CK: sucrose control group.

Changes in the symbiotic bacterial community composition and abundance were next analyzed at the phylum level. Second-instar larvae treated with Cry1B and those treated with sucrose for 24 or 48 h had similar dominant phyla, including Firmicutes (*p* = 0.742 and 0.036), Proteobacteria (*p* = 0.786 and 0.1215), Bacteroides (*p* = 0.556 and 0.2783), and Actinobacteria (*p* = 0.5425 and 0.1431) (*p* value represents a significant difference between the two components). The change trend for dominant bacteria abundance was different between the groups treated for 24 h and 48 h (Figure 3A). A comparison of each treatment group to the control group revealed no significant changes in the abundance of *P. japonica* bacterial community members in response to 0.25 or 0.5 mg/mL Cry1B treatment for 24 or 48 h. The only significant difference was in the abundance of Firmicutes among second-instar larvae in the control group (47.78%) compared to those treated with 1.0 mg/mL Cry1B for 48 h (96.26%) (*p* = 0.03607). Some low-abundance bacteria also responded to treatment with exogenous Bt protein, although most taxa showed no significant changes in abundance compared to the control group at either treatment duration or at any concentration of Cry1B. Only two low-abundance taxa were significantly differentially abundant in any treatment group compared to the control: Gemmatimonadetes (*p* = 0.036) (15.13% in C3 and 4.76% in CK) in *P. japonica* treated with 1.0 mg/mL Cry1B for 24 h and Patescibacteria (*p* = 0.023) (C3: 0.34%, CK: 6.83%) in those treated with 1.0 mg/mL Cry1B for 48 h (Figure 4A,B,D).

Among third-instar larvae, the dominant phyla in the sucrose control group were consistent between those treated for 24 and 48 h: Firmicutes, Proteobacteria, Bacteroides, and Actinobacteria. The abundance of each dominant phylum did not show consistent changes along with increases in the Cry1B treatment concentration (Figure 3B). For example, after 24 h treatment with 0.25, 0.5, and 1.0 mg/mL Cry1B, the abundance of Firmicutes (95.73%, 91.46%, and 93.40%, respectively) and Proteobacteria (3.23%, 1.78%, and 5.55%, respectively) first decreased and then increased. In contrast, *P. japonica* treated for 48 h showed consistent decreases in the abundance of Firmicutes as the Cry1B concentration increased from 0.25 to 0.5 and 1.0 mg/mL Cry1B (94.37%, 83.29%, and 78.31%, respectively). However, differences in the abundance of the four dominant phyla at 24 and 48 h of treatment compared to the control were not statistically significant (*p* = 0.721 and 0.691, respectively, for Firmicutes; 0.227 and 0.698, respectively, for Protobacteria; 0.341 and 0.396, respectively, for Bacteroides). There were no other notable changes in the abundance of non-dominant taxa; Deinococcus-Thermus abundance was only markedly different between treatment groups (C2_1D with C3_1D) (Figure 4C). 

### 3.4. Effects of Cry1B Treatment on P. japonica Microbiota at the Genus Level

**Figure 5:**
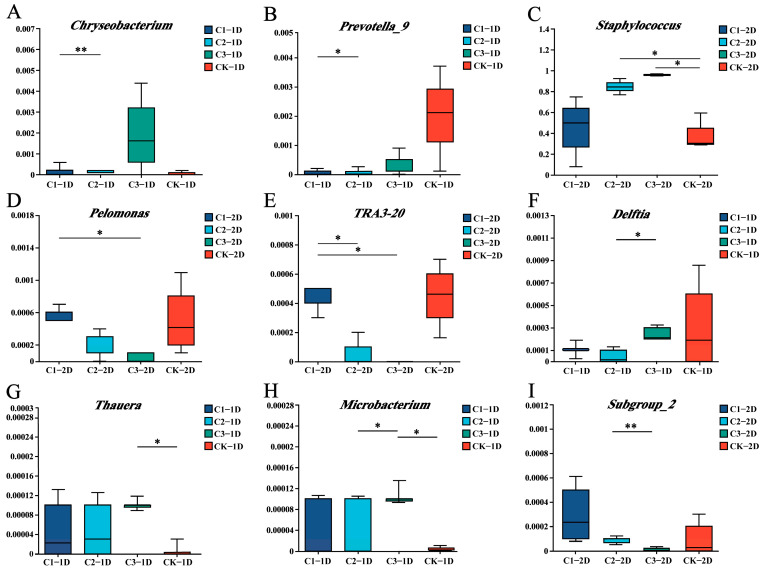
Relative abundance of endophytic bacterial genera among *Propylea japonica* treated with Cry1B or a sucrose control: (**A**–**E**) Differences in the bacterial communities of second-instar larvae treated for (**A**,**B**) 24 h (2L1D) or (**C**–**E**) 48 h (2L2D). (**F**–**I**) Differences in the bacterial communities of third-instar larvae treated for (**F**–**H**) 24 h (3L1D) or (**I**) 48 h (3L2D). Data are shown only for genera with statistically significant differences between groups. * *p* ≤ 0.05, ** *p* ≤ 0.01 (one-way analysis of variance). C1: 0.25 mg/mL Cry1B; C2: 0.5 mg/mL Cry1B; C3: 1.0 mg/mL Cry1B; and CK: sucrose control group.

Further analyses were conducted to assess differences in the composition and abundance of commensal bacteria at the genus level among second- and third-instar *P. japonica* larvae treated with several concentrations of Cry1B for 24 and 48 h. Among the second-instar larvae, the dominant bacterial genera were consistent between those treated with Cry1B and those in the sucrose control group. The dominant genera were *Staphylococcus*, *Ralstonia*, *Lactobacillus*, and *Muribaculaceae*. At 24 h of treatment, the Cry1B treatment concentration was inversely correlated with Staphylococcus abundance (89.17%, 84.74%, and 56.15% in those treated with 0.25, 0.5, and 1.0 mg/mL Cry1B, respectively), although the differences were not statistically significant (*p* = 0.692). In contrast, *Ralstonia*, *Muribaculaceae*, and *Lactobacillus* all increased in abundance along with the Cry1B concentration, but these differences were not significant either (*p* = 0.484, 0.596, and 0.471, respectively). After treatment for 48 h, those treated with higher Cry1B concentrations showed statistically insignificant decreases in *Ralstonia* (*p* = 0.155), *Lactobacillus* (*p* = 0.383), and *Muribaculaceae* (*p* = 0.309). Only one dominant taxon, *Staphylococcus* aureus, showed statistically significant differences in *P. japonica* treated with 0.5 mg/mL or 1.0 mg/mL Cry1B compared to the control group (*p* = 0.028 for both groups). Several low-abundance taxa were significantly different between Cry1B treatment groups, but the differences were not significant in comparison to the control group. For example, there were statistically significant differences in *Chryseobacterium* and *Prevotella_9* abundance between *P. japonica* treated with different Cry1B concentrations for 24 h (*p* = 0.012 and 0.030, respectively). Among second-instar larvae treated for 48 h, there were significant differences between Cry1B treatment groups with respect to *Pelomonas* and *TRA3-20* abundance (*p* = 0.027 and 0.011, respectively) (Figure 3C and Figure 5A–E). 

Among third-instar *P. japonica* larvae, the predominant genera were *Staphylococcus* and *Ralstonia* among both those treated with Cry1B and those treated with sucrose for 24 or 48 h. At 24 h, there were some alterations in the abundance of *Staphylococcus* (*p* = 0.744) and *Ralstonia* (*p* = 0.270) along with increased Cry1B concentrations, but there were no significant differences compared to the control group. At 48 h, increased Cry1B concentrations were associated with alterations in the abundance of the dominant genera *Staphylococcus* (*p* = 0.717), *Ralstonia* (*p* = 0.054), *Acinetobacter* (*p* = 0.524), and *Lactobacillus* (*p* = 0.269), but none of the differences were statistically significant compared to the control group. Several low-abundance taxa did show significant differences compared to the control. For example, there were significant differences in *Thauera* (*p* = 0.044) and *Microbacterium* (*p* = 0.031) between third-instar larvae treated with a high Cry1B concentration (1.0 mg/mL) for 24 h compared to the control. In addition, there were statistically significant differences in the abundance of *Subgroup_2* (*p* = 0.022) and *Delftia* (*p* = 0.022) between the Cry1B treatment groups at 48 and 24 h, respectively, but not between the Cry1B and control groups (Figure 3D and Figure 5F–I). 

**Figure 6:**
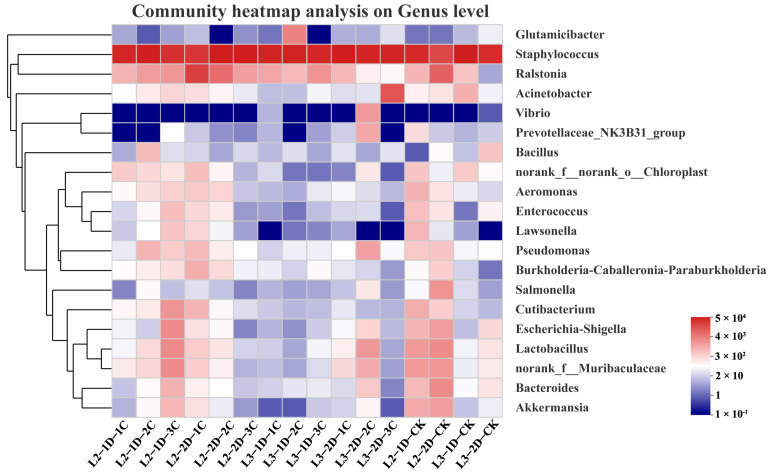
Relative abundance of the 20 most abundant genera across samples. C1: 0.25 mg/mL Cry1B; C2: 0.5 mg/mL Cry1B; C3: 1.0 mg/mL Cry1B; and CK: sucrose control group.

A comprehensive analysis of all samples at the genus level yielded 20 genera with high abundance. *Staphylococcus* was the most abundant genus in all samples, followed by *Ralstonia*. A heatmap analysis showed that the compositions of dominant bacteria were largely consistent between the Cry1B treatment groups and the control. Compared with the control group, the largest changes in abundance were in the genera *Acinetobacter*, *Vibrio*, and *Glutamicibacter* (Figure 6). 

## 4. Discussion

*P. japonica* is a common arthropod with high temperature resistance and strong predation abilities, and it is the dominant natural enemy in many farmland ecosystems and preys on cotton aphids, plant hoppers, cotton bollworms, and other pests [20]. It comes in contact with Bt proteins both directly and indirectly during insect predation, and is therefore often used as an indicator insect to assess the potential risks of transgenic crops [39]. Numerous studies have examined the effects of Bt proteins on non-target insect growth, development, enzyme activity, and expression of detoxification metabolism genes. For instance, one study examined the pupation rate, emergence rate, 7 d larva weight, and sexual ratio of *Harmonia axyridis* (Pallas) (Coleoptera: Coccinellidae) to assess the environmental risks of the Bt proteins Cry1Ac, Cry2Ab, Cry1Ca, and Cry1F [40]. Other studies focusing on the food chain have shown that aphids are not affected by Bt proteins, eliminating the impact of prey on the natural enemy *Eupeodes americanus* (Wiedemann) (Diptera: Syrphidae) [41]. In addition to the evaluation of Bt safety in natural enemies, it is also important to consider the responses of non-target insect microbiota. Micro-organisms are integral to ecological functions; endophytic bacteria play crucial roles in parasitism, reproduction, resistance to foreign pathogens, and even key nutrient synthesis [21]. Therefore, the study of endophytic bacteria is of great significance to understanding insect mechanisms of action [42]. Endophytic bacteria differ from rhizosphere bacteria in numerous ways. For example, the indoor feeding conditions of insects cause endophytic bacteria to be in a stable state [43]. As a result, insect endophytic bacteria can be used to assess the environmental risks of Bt protein to non-target insects [44]. However, the effects of the Bt protein Cry1B on the bacterial community of *P. japonica* have not been well characterized. In the present study, the *P. japonica* bacterial community was used as an indicator of Bt protein environmental risks. Investigating the effects of Bt proteins on the composition and structure of commensal bacteria in insects is crucial to evaluations of transgenic crop safety. 

Here, we found that the *P. japonica* larval bacterial community was dominated at the phylum level by Firmicutes and Proteobacteria and at the genus level by Staphylococcus and Ralstonia. The abundance levels of the dominant bacterial taxa were altered by treatment with different Bt concentrations and by treatment for different lengths of time, but the dominant species remained the same. These results were consistent with a previous study analyzing the microflora of non-target insect fourth-instar larvae treated with Bt protein. In that study, the most abundant phyla in *P. japonica* were Firmicutes, Proteobacteria, and Actinobacteria, and the most abundant genera were *Staphylococcus*, *Ralstonia*, *Acinetobacter*, and *Lactobacillus* [45]. Another study examined the effects of conventional feed and food containing Cry2Ab on the *P. japonica* bacterial community [44]. There were no significant differences in the microbial community diversity or structure between Cry-protein-treated groups; furthermore, the results showed that Cry2Ab had no effect on *P. japonica* development or reproduction. That study also showed that the most abundant bacterial taxa at the phylum and genus levels were Firmicutes and *Staphylococcus*, respectively; the differences in the total number of bacteria were not significant at the larval stage; and Bt protein had a greater influence on the abundance of various bacterial genera than on community membership [44]. A comparison of previous studies on bacterial community diversity revealed that different Bt proteins had varying effects on the diversity of endophytic bacteria in *P. japonica*. These prior experimental results are consistent with the findings of the present study, demonstrating that Bt protein treatment did not significantly alter the dominant bacterial species or membership of endophytic bacteria in *P. japonica*. 

The comparison of the microbial communities in insects treated with different concentrations of Bt protein presented here showed differences in the abundance of several genera. The abundance of most taxa did not change significantly after treatment with low Bt concentrations for different lengths of times, and very few taxa showed significant changes at high concentrations of Bt protein. However, there were significant differences between treatment groups in the abundance of taxa such as *Chryseobacterium*, *Prevotella-9* (2L1D), *Pelomonas*, *TRA3-20* (2L2D), and *Subgroup-2* (3L2D). Only a few of the treatment groups showed significant differences compared to the control. Further analysis showed that differences in community structure between those treated with a high concentration of Cry1B (1.0 mg/mL) and those treated with sucrose were primarily due to altered abundance of *Staphylococcus* (2L2D), *Thauera*, and *Microbacterium* (3L1D). Comparisons of α-diversity indices (namely ACE and Shannon) were consistent with the results at the genus level, with significant differences in the control group compared to the high-Cry1B treatment. Previous studies have examined the effects of Cry1B treatment on *P. japonica* growth, enzyme activity, and expression of detoxification metabolism genes. Those studies showed no significant effects of Cry1B at three different concentrations on the biological performance of *P. japonica*, and only the highest Cry1B concentration significantly affected enzyme activity and the expression of detoxification metabolism genes [20]. Some researchers have also found that changes caused by genetic engineering in maize pollen may not lead to biological effects in *P. japonica* [46]. However, silkworm larvae treated with a high concentration of Bt pollen show significant differences in weight after molting compared with a control group [47]. In *Pardosa pseudoannulata*, individuals treated with Bt protein have lower microbial species abundance than the control group, but higher species uniformity [48]. Another study in the non-target pest *Nilaparvata lugens* (Stal) (Hemiptera: Delphacidae) (using the Biolog-Eco method) found that exposure to transgenic Cry1Ab (KMD1/KMD2) had no obvious negative impact on intestinal microbial community diversity [49]. A study examining the microbial community composition of honeybee (*Apis mellifera ligustica*) individuals in different Bt treatment groups used a denaturing gradient gel electrophoresis (DGGE) map, which revealed some differences in bacterial community structure between those treated with non-transgenic cotton pollen and those treated with a sucrose control [50]. Summarizing the above, it can be found that differences that were statistically significant may have been due to stimulation with high concentrations of exogenous protein. Different types of Bt proteins could have varying effects on non-target insect bacterial communities. The abundance of endophytic bacteria in insects is obviously related to the concentration of Bt protein. However, the concentrations of Bt protein that are expressed in the field by transgenic insect-resistant crops are well below the minimum concentrations used in the present study [31]. It is highly unlikely that insects would be exposed to Bt proteins at concentrations as high as 1.0 mg/mL in the field, suggesting that environmentally relevant concentrations of Cry1B may not have a significant effect on the predator *P. japonica* [47]. Many studies have shown that predators are only affected when Bt-sensitive herbivores are used as prey, with no evidence of direct toxic effects [41]. Field survey data have also confirmed that the abundance and diversity of target and non-target insects are similar in Bt and non-Bt fields; this again suggests that Bt may not be toxic to non-target insects [51].

During insect development, many commensal micro-organisms are inherited through vertical transmission, although some beneficial commensal bacteria are also acquired through contact with the external environment [23]. These commensal bacteria can contribute to host insect development and reproduction by altering host mating behavior, enhancing tolerance or resistance to pathogenic fungi and natural enemies, or influencing the host species population dynamics and genetic diversity [52,53]. Through the previous research results, we can speculate on the function of endophytic bacteria in *P. japonica*. For example, *Staphylococcus* is also a prominent component of the *Apriona germari* (Hope) (Coleoptera: Cerambycidae) larval microbial communities and plays a positive role in host growth and development [54]. The largest differences in microbial community structure between Cry1Ac-resistant and Cry1Ac-sensitive strains are in the phylum Firmicutes [55]. In the present study, the most abundant Firmicutes genus in *P. japonica* was *Staphylococcus*, which increased in abundance after feeding on exogenous Bt protein. We therefore speculate that the increased abundance of *Staphylococcus* promoted host resistance to adverse reactions to Cry1B. *Staphylococcus* remained the dominant genus across treatment groups with different concentrations of Bt protein and treatment lengths; the relative abundance of this genus did not consistently change with increased host age, and there were no significant fluctuations in the abundance. Therefore, it is inferred that the abundance of *Staphylococcus* has nothing to do with the host age, which indicates that *Staphylococcus* may be vertically inherited in *P. japonica*. In other related studies, compared with the control, the abundance of Botulinum in the *P. japonica* and *Plutella xylostella* fed with Bt changed irregularly, indicating that *Botulinum* does not respond to Bt protein; changes in *Botulinum* abundance are therefore only related to the environmental conditions in which the host insects are located [56]. Here, treatment with Bt protein decreased the abundance of *Bacillus* and *Stenotrophomonas*, indicating that these species may not have contributed to host Cry1B resistance. Conversely, *Enterococcus* abundance increases along with the larval growth stage of Bt-treated *P. japonica*, suggesting that *Enterococcus* may increase Bt protein resistance [29]. In this study, the possibility of changes in several dominant genera was obtained based on the above-mentioned series of research rules. For example, *P. japonica* treated with Cry1B showed significant increases in *Ralstonia* abundance, leading to the speculation that this genus may have resistance to Cry1B. Levels of *Acinetobacter* and *Lactobacillus* did not show consistent changes among *P. japonica* treated with Bt protein compared with the control group, and we therefore speculated that their abundance was related to environmental conditions rather than Bt protein exposure. At present, some studies have found that Enterobacteriaceae can prolong the life of insects [57]; it can significantly improve the adult size, pupa weight, survival rate and mating competitiveness [58]; it can significantly increase the weight and body length of the host and significantly shorten its growth cycle [59]; and it was also found that the nutritional metabolism of *Rhynchophorus ferrugineus* (Olivier) (Coleoptera: Curculionidae) was significantly affected and the immune defense function was also significantly reduced after the main endophyte *Lactobacillus* was completely reduced or eliminated [60,61]. When the core bacterium *Lactobacillus* is absent from the intestine of *Drosophila melanogaster*, its pupation time is prolonged and its wings become smaller [62]; *Pseudomonas protegens*, a rare bacterium in *Nasonia vitripennis*, can improve the drug resistance of this parasitic wasp by metabolizing atrazine [63]. In this study, the relative abundance of *Salmonella* (of Enterobacteriaceae), *Lactobacillus*, and *Pseudomonas* all changed in different degrees, speculating that the relative abundance changes in these endophytes may have an impact on the growth and development, immune defense, and other aspects of *P. japonica*.

In this study, *P. japonica* was treated with Cry1B protein for a short time, and then the effect of this protein on the bacterial community of *P. japonica* was analyzed. However, it is important to note that long-term exposure of *P. japonica* to Bt proteins in the field may yield different results, and the impact of a single strain on the growth and development of *P. japonica* remains unclear. Future studies are necessary to verify the influence of endophytic bacteria on *P. japonica* biology and to evaluate microbial community changes in response to stressors and other external pressures found in the farmland environment. In addition, this study was based on sucrose solution as the control, and no other protein was added as the supplementary control. The differences in the influence of common proteins and Bt proteins on the biology of *P. japonica* need further study.

## 5. Conclusions

In this study, a high-throughput sequencing method was used to compare the bacterial communities of *P. japonica* given Cry1B protein or a sucrose control. Cry1B treatment will have some influence on endophytic bacteria in this non-target insect. Cry1B ingestion did not change the microbial community membership in *P. japonica*, but altered the abundance of some dominant and some less abundant bacteria. Together with data from prior studies, these results suggested that high Bt protein concentrations significantly altered the bacterial communities in *P. japonica* without affecting insect biological performance. The precise effects of changes in the abundance of some endophytic bacteria require further evaluation. Furthermore, field studies should be conducted to directly assess the potential individual and synergistic impacts of large-scale Bt crop cultivation on non-target organisms. This experiment provides data support for the safety evaluation of genetically modified crops.

## Figures and Tables

**Table 1:** Sequencing depth and α-diversity index values at different taxonomic levels for each sample.

Sample Name	Original Tags	High Quality Tags	OUTs	α Diversity Index				
				Shannon	Simpson	Ace	Chao	Coverage
L2_1D_C1	66,516	66,516	346	2.9537	0.2055	593.04	600.54	0.9991
L2_1D_C2	61,032	60,538	331	1.0529	0.6660	340.31	342.44	0.9981
L2_1D_C3	60,905	59,015	394	2.7374	0.3999	363.07	362.32	0.9991
L2_1D_CK	60,664	57,962	458	0.9368	0.7562	305.00	301.59	0.9984
L2_2D_C1	63,288	64,417	600	2.6984	0.3527	337.38	350.43	0.9995
L2_2D_C2	60,854	60,854	236	0.9639	0.6477	245.19	245.53	0.9986
L2_2D_C3	54,419	54,419	191	0.3289	0.9000	232.71	234.11	0.9979
L2_2D_CK	48,682	48,682	532	2.6801	0.2359	575.59	563.47	0.9968
L3_1D_C1	46,717	46,717	290	0.8338	0.6178	402.64	351.45	0.9968
L3_1D_C2	48,830	48,830	249	0.8679	0.5559	404.96	345.06	0.9965
L3_1D_C3	45,021	45,021	297	0.8438	0.6575	339.16	322.41	0.9973
L3_1D_CK	56,730	56,730	281	0.9826	0.5119	351.04	336.39	0.9971
L3_2D_C1	57,147	57,147	406	0.9641	0.6492	456.31	432.79	0.9966
L3_2D_C2	57,909	57,909	451	1.7358	0.4001	576.14	487.87	0.9963
L3_2D_C3	55,863	55,863	153	0.8632	0.5460	308.40	211.03	0.9980
L3_2D_CK	43,716	43,716	662	1.7741	0.4718	746.87	738.36	0.9954

Note: L2 represents the second-instar larvae, L3 represents the third-instar larvae. 1D represents one day (24 h) of treatment, 2D represents two days (48 h) of treatment, C1 represents 0.25 mg/mL, C2 represents 0.5 mg/mL, and C3 represents 1.0 mg/mL. CK represents the control group. The same below.

## Data Availability

The data presented in this study are available on request from the corresponding author. The data are not publicly available due to sensitivity of research content.

## References

[1] Tilgam: J., Kumar K., Jayaswal D., Choudhury S., Kumar A., Jayaswall K., Saxena A.K. (2021). Success of microbial genes based transgenic crops: Bt and beyond Bt. Mol. Biol. Rep..

[2] Řezbová H., Škubna O. (2012). The Role of Transgenic Crops in the Future of Global Food and Feed. AGRIS-Line Pap. Econ. Inform..

[3] Parmar N., Singh K.H., Sharma D., Singh L., Kumar P., Nanjundan J., Khan Y.J., Chauhan D.K., Thakur A.K. (2017). Genetic engineering strategies for biotic and abiotic stress tolerance and quality enhancement in horticultural crops: A comprehensive review. 3 Biotech.

[4] Kumar K., Gambhir G., Dass A., Tripathi A.K., Singh A., Jha A.K., Yadava P., Choudhary M., Rakshit S. (2020). Genetically modified crops: Current status and future prospects. Planta.

[5] Bigler F., Candolfi M.P. Moving Through the Tiered and Methodological Framework for NonTarget Arthropod Risk Assessment of Transgenic Insecticidal Crops. Proceedings of the 9th International Symposium on the Biosafety of Genetically Modified Organisms.

[6] Marvier M. (2002). Improving risk assessment for nontarget safety of transgenic crops. Ecol. Appl..

[7] Romeis J., Meissle M., Alvarez-Alfageme F., Bigler F., Bohan D.A., Devos Y., Malone L.A., Pons X., Rauschen S. (2014). Potential use of an arthropod database to support the non-target risk assessment and monitoring of transgenic plants. Transgenic Res..

[8] Romeis J., Meissle M. (2011). Non-target risk assessment of Bt crops—Cry protein uptake by aphids. J. Appl. Entomol..

[9] Waseem M., Khan M.B., Ahmad F., Farooq S., Id M.H. (2020). The influence of transgenic (Bt) and non- transgenic (non-Bt) cotton mulches on weed dynamics, soil properties and productivity of different winter crops. PLoS ONE.

[10] Tayyib M., Sohail A., Murtaza A., Jamil F.F. (2005). Efficacy of some new-chemistry inseticides for controlling the sucking insect peats and mites on cotton. Pak. Entomol..

[11] Chen Y., Li Y., Zhou M., Cai Z., Tambel L.I.M., Zhang X., Chen Y., Chen D. (2019). Nitrogen deficit decreases seed Cry1Ac endotoxin expression in Bt transgenic cotton. Plant Physiol. Biochem..

[12] Bravo A., Gill S.S., Soberón M. (2007). Mode of action of Bacillus thuringiensis Cry and Cyt toxins and their potential for insect control. Toxicon.

[13] Glare T.R., O’Callaghan M. (2000). Bacillus thuringiensis: Biology, Ecology and Safety.

[14] Azizoglu U., Jouzani G.S., Yilmaz N., Baz E., Ozkok D. (2020). Genetically modified entomopathogenic bacteria, recent developments, benefits and impacts: A review. Sci. Total Environ..

[15] Schnepf E., Crickmore N., Van Rie J., Lereclus D., Baum J., Feitelson J., Zeigler D.R., Dean D.H. (1998). Bacillus thuringiensis and Its Pesticidal Crystal Proteins. Microbiol. Mol. Biol. Rev..

[16] Mehmood Y., Zaheeruddin F., Bakhsh K., Anjum M.B., Ahmad M. (2012). Impact of Bt. cotton varieties on productivity: Evidence from district Vehari, Pakistan. J. Agric. Soc. Sci..

[17] Lu Y., Wu K., Jiang Y., Guo Y., Desneux N. (2012). Widespread adoption of Bt cotton and insecticide decrease promotes biocontrol services. Nature.

[18] Chen Y., Lai F.X., Sun Y.Q., Hong L.Y., Tian J.C., Zhang Z.T., Qiang F.U. (2015). Cry1Ab rice does not impact biological characters and functional response of *Cyrtorhinus lividipennis* preying on *Nilaparvata lugens* eggs. J. Integr. Agric..

[19] Yang H., Peng Y., Tian J., Wang J., Hu J., Song Q., Wang Z. (2017). Review: Biosafety assessment of Bt rice and other Bt crops using spiders as example for non-target arthropods in China. Plant Cell Rep..

[20] Li Y., Diao F., Zhu X., Wang L., Zhang K., Li D., Ji J., Niu L., Gao X., Luo J. (2022). Transgenic cotton expressing Cry1B protein has no adverse effect on predatory insect *Propylea Japonica*. Ecotoxicol. Environ. Saf..

[21] McCutcheon J.P., Moran N.A. (2010). Functional convergence in reduced genomes of bacterial symbionts spanning 200 My of evolution. Genome Biol. Evol..

[22] Dillon R.J., Vennard C.T., Buckling A., Charnley A.K. (2010). Diversity of locust gut bacteria protects against pathogen invasion. Ecol. Lett..

[23] Sharon G., Segal D., Ringo J.M., Hefetz A., Zilber-Rosenberg L., Rosenberg E. (2010). Commensal bacteria play a role in mating preference of *Drosophila melanogaster*. Proc. Natl. Acad. Sci. USA.

[24] Brand J.M., Bracke J.W., Markovetz A.J., Wood D.L., Browne L.E. (1975). Production of verbenol pheromone by a bacterium isolated from bark beetles. Nature.

[25] Gaugler R. (2002). Entomopathogenic Nematology.

[26] König H. (2006). Bacillus species in the intestine of termites and other soil invertebrates. J. Appl. Microbiol..

[27] Wenzel M., Schönig I., Berchtold M., Kämpfer P., König H. (2002). Aerobic and facultatively anaerobic cellulolytic bacteria from the gut of the termite *Zootermopsis angusticollis*. J. Appl. Microbiol..

[28] Zhu F.J. (2018). Effects of Bt Proteins Cry1Ab, Cry1F and Cry3B on Earthworm and Intestinal Microorganisms.

[29] Wu L.K. (2018). Studies of Biosafety of Bt Proteins on Propylea Japonica Using the Transcriptome and 16s Sequencing Technology.

[30] Singh A.K., Dubey S.K. (2016). Current trends in Bt crops and their fate on associated microbial community dynamics: A review. Protoplasma.

[31] Li Y., Zhang X., Chen X., Romeis J.R., Yin X., Peng Y. (2015). Consumption of Bt rice pollen containing Cry1C or Cry2A does not pose a risk to *Propylea japonica* (Thunberg) (Coleoptera: Coccinellidae). Sci. Rep..

[32] Romeis J., Hellmich R.L., Candolfi M.P., Carstens K., Schrijver A.D., Gatehouse A., Herman R.A., Huesing J.E., Mclean M.A., Raybould A. (2011). Recommendations for the design of laboratory studies on non-target arthropods for risk assessment of genetically engineered plants. Transgenic Res..

[33] Khan I.A., Wan F.H. (2008). Life table of *Propylea japonica* Thunberg (Coleoptera, Coccinellidae) fed on *Bemisia tabaci* (Gennadius) (Homoptera, Aleyrodidae) biotype B prey. Sarhad. J. Agric..

[34] Zhang L., Li S., Luo J., Du P., Wu L., Li Y., Zhu X., Wang L., Zhang S., Cui J. (2020). Chromosome-level genome assembly of the predator *Propylea japonica* to understand its tolerance to insecticides and high temperatures. Mol. Ecol. Resour..

[35] Shelton A.M., Zhao J.Z., Roush R.T. (2002). Economic, ecological, food safety, and social consequences of the deployment of bt transgenic plants. Annu. Rev. Entomol..

[36] Alvarez-Alfageme F., Bigler F., Romeis J. (2011). Laboratory toxicity studies demonstrate no adverse effects of Cry1Ab and Cry3Bb1 to larvae of Adalia bipunctata (Coleoptera: Coccinellidae): The importance of study design. Transgenic Res..

[37] Knight K., Head G., Rogers J. (2013). Season-long expression of Cry1Ac and Cry2Ab proteins in Bollgard II cotton in Australia. Crop Prot..

[38] Meissle M., Romeis J. (2018). Transfer of Cry1Ac and Cry2Ab proteins from genetically engineered Bt cotton to herbivores and predators. Insect Sci..

[39] Ramirez-Romero R., Desneux N., Chaufaux J., Kaiser L. (2008). Bt-maize effects on biological parameters of the non-target aphid *Sitobion avenae* (Homoptera : Aphididae) and Cry1Ab toxin detection. Pestic. Biochem. Physiol..

[40] Ali I., Zhang S., Iqbal M., Ejaz S., Cui J.J. (2017). Trypsinized Cry1Fa and Vip3Aa have no detrimental effects on the adult green lacewing *Chrysopa pallens* (Neuroptera: Chrysopidae). Appl. Entomol. Zool..

[41] Tian J.C., Yao J., Long L.P., Romeis J., Shelton A.M. (2015). Bt crops benefit natural enemies to control non-target pests. Sci. Rep..

[42] Kremer R.J., Means N.E. (2009). Glyphosate and glyphosate-resistant crop interactions with rhizosphere microorganisms. Eur. J. Agron..

[43] Dong L., Meng Y., Wang J., Sun G. (2016). Effects of Transgenic Bt + CpTI cotton on the abundance and diversity of rhizosphere ammonia oxidizing bacteria and archaea. J. Environ. Biol..

[44] Zhang S., Luo J., Jiang W., Wu L., Zhang L., Ji J., Wang L., Ma Y., Cui J. (2019). Response of the bacterial community of *Propylea japonica* (Thunberg) to Cry2Ab protein. Environ. Pollut..

[45] Zhao C., Wu L., Luo J., Niu L., Wang C., Zhu X., Wang L., Zhao P., Zhang S., Cui J. (2020). Bt, Not a Threat to *Propylea japonica*. Front. Physiol..

[46] Wang Y., Liu Q., Song X., Yang X., Han L., Romeis J., Li Y. (2022). Unintended changes in transgenic maize cause no nontarget effects. Plants People Plane.

[47] Niu L., Ma Y., Mannakkara A., Zhao Y., Ma W., Lei C., Chen L. (2013). Impact of single and stacked insect-resistant Bt-cotton on the honey bee and silkworm. PLoS ONE.

[48] Xiong H., Li D., Liang G.H., Wang Z., Wei B.Y. (2018). Effect of Bt protein on intestinal bacterial diversity of the *Pardosa pseudoannulata*. J. Hunan Agric. Univ..

[49] Han L., Jiang X., Peng Y. (2016). Potential resistance management for the sustainable use of insect-resistant genetically modified corn and rice in China. Curr. Opin. Insect Sci..

[50] Jiang W.Y., Dai P.L., Zhang Y.J., Zhou T., Lin Y., Shu C.L., Zhang J. (2010). Effect of transgenic cotton with Cry1Ac gene on intestinal baterial community of *Apis mellifera ligustica*. Chin. J. Appl. Environ. Biol..

[51] Romeis J., Meissle M., Bigler F. (2006). Transgenic crops expressing Bacillus thuringiensis toxins and biological control. Nat. Biotechnol..

[52] Crotti E., Balloi A., Hamdi C., Sansonno L., Marzorati M., Gonella E., Favia G., Cherif A., Bandi C., Alma A. (2012). Microbial symbionts: A resource for the management of insect-related problems. Microb. Biotechnol..

[53] Tsuchida T., Koga R., Horikawa M., Tsunoda T., Maoka T., Matsumoto S., Simon J.C., Fukatsu T. (2010). Symbiotic bacterium modifies aphid body color. Science.

[54] Zhang W., He Z., Deng X., Yin Y. (2004). Variety and distribution of intestinal flora of *Apriona germari* (hope) larvae. J. Southwest Agric. Univ..

[55] Jiang W.Y., Liang G.M., Lin Y., Shu C.L., Song F.P., Zhang J. (2010). Comparison of midgut bacterial community between Bt resistant and sensitive *Helicoverpa armiger*. J. Microbiol..

[56] Dai N.J. (2014). Diversity and Synergistic Effect with BT of the Intestine Bacterial Community of Plutella Xylostella Lavae.

[57] Behar A., Jurkevitch E., Yuval B. (2008). Bringing back the fruit into fruit fly-bacteria interactions. Mol. Ecol..

[58] Zhang Q.P., Cai B., Wang X., Liu J., Lin R., Hua H., Zhang C., Yi X., Song Q., Ji J. (2021). Manipulation of Gut Symbionts for Improving the Sterile Insect Technique: Quality Parameters of *Bactrocera dorsalis* (Diptera: Tephritidae) Genetic Sexing Strain Males After Feeding on Bacteria-Enriched Diets. J. Econ. Entomol..

[59] Zhang Q., Wang S.X., Zhang K., Zhang W., Liu R., Zhang R.L., Zhang Z. (2021). *Enterobacter hormaechei* in the intestines of housefly larvae promotes host growth by inhibiting harmful intestinal bacteria. Parasit Vectors.

[60] Muhammad A., Habineza P., Ji T.L., Hou Y.M., Shi Z.H. (2019). Intestinal Microbiota Confer Protection by Priming the Immune System of Red Palm Weevil *Rhynchophorus ferrugineus* Olivier (Coleoptera: Dryophthoridae). Front. Physiol..

[61] Habineza P., Muhammad A., Ji T.L., Xiao R., Yin X.Y., Hou Y.M., Shi Z.H. (2019). The Promoting Effect of Gut Microbiota on Growth and Development of Red Palm Weevil, *Rhynchophorus ferrugineus* (Olivier) (Coleoptera: Dryophthoridae) by Modulating Its Nutritional Metabolism. Front. Microbiol..

[62] Shin S.C., Kim S.H., You H., Kim B., Kim A.C., Lee K.A., Yoon J.H., Ryu J.H., Lee W.J. (2011). *Drosophila* Microbiome Modulates Host Developmental and Metabolic Homeostasis via Insulin Signaling. Science.

[63] Wang G.H., Berdy B.M., Velasquez O., Jovanovic N., Alkhalifa S., Minbiole K.P.C., Brucker R.M. (2020). Changes in Microbiome Confer Multigenerational Host Resistance after Sub-toxic Pesticide Exposure. Cell Host Microbe.

